# Comorbidity in Midlife and Cancer Outcomes

**DOI:** 10.1001/jamanetworkopen.2025.3469

**Published:** 2025-04-07

**Authors:** Jessica A. Lavery, Paul C. Boutros, Chaya S. Moskowitz, Lee W. Jones

**Affiliations:** 1 Memorial Sloan Kettering Cancer Center, New York, New York; 2 Institute for Precision Health, University of California, Los Angeles; 3 Jonsson Comprehensive Cancer Center, University of California, Los Angeles; 4 Department of Urology, University of California, Los Angeles; 5 Department of Human Genetics, University of California, Los Angeles; 6 Weill Cornell Medicine, New York, New York

## Abstract

**Importance:**

Comorbidities in midlife are common but how these conditions are associated with cancer outcomes is poorly understood.

**Objective:**

To investigate the association between different comorbidities and risk of incident cancer and cancer mortality.

**Design, Setting, and Participants:**

This cohort study is a secondary analysis of the prospective Prostate, Lung, Colorectal, and Ovarian (PLCO) screening trial conducted at 10 PLCO screening centers across the US. Participants included adults aged 55 to 74 years without a history of cancer enrolled between 1993 and 2001. Statistical analysis was performed from June 2023 to December 2024.

**Exposures:**

Self-reported history of 12 comorbid conditions classified into 5 distinct classifications guided by World Health Organization categorization.

**Main Outcome and Measures:**

Outcomes included risk of all cancers combined, risk of 19 individual cancer types, and cancer mortality. Multivariable Cox proportional hazards models were used to estimate the association between comorbidity classifications and cancer outcomes.

**Results:**

Among 128 999 participants included in the analysis, 330 (0.3%) were American Indian, 5414 (4.2%) were Asian or Pacific Islander, 6704 (5.2%) were non-Hispanic Black, and 114 073 (88.4%) were non-Hispanic White; 64 171 (49.7%) were male; and the median (IQR) age was 62 (58-66) years. After a median (IQR) follow-up of 20 (19-22) years, the risk of any incident cancer was significantly higher for individuals with a history of respiratory (hazard ratio [HR], 1.07 [95% CI, 1.02-1.12]) and cardiovascular conditions (HR, 1.02 [95% CI, 1.00-1.05]). History of each comorbid condition evaluated was significantly associated with incidence of at least 1 cancer type. The strongest association was between history of liver conditions and risk of liver cancer (HR, 5.57 [95% CI, 4.03-7.71]), whereas metabolic conditions (obesity or type 2 diabetes) were significantly associated with higher risk of 9 cancer types and lower risk of 4 cancer types. Respiratory (HR, 1.19 [95% CI, 1.11-1.28]), cardiovascular (HR, 1.08 [95% CI, 1.04-1.13]), and metabolic (HR, 1.09 [95% CI, 1.05-1.14]) conditions were positively associated with a higher hazard of cancer death.

**Conclusions and Relevance:**

In this cohort study of 128 999 adults without a history of cancer, comorbidities in midlife were associated with the overall risk of cancer and more strongly associated with risk of multiple individual cancer types, with the direction of association differing across cancer types. These results may inform clinical management of patients at risk for cancer.

## Key Points

**Question:**

Is there an association between comorbidity in midlife with cancer outcomes?

**Findings:**

In this cohort study including 128 999 adults without a history of cancer, comorbidities in midlife were associated with risk of any cancer and more strongly associated with risk of multiple individual cancer types, with the direction of association differing across cancer types.

**Meaning:**

These findings may inform clinical management of individuals who are at risk of cancer.

## Teaser

This cohort study examines whether there is an association between different comorbidities and risk of incident cancer and cancer mortality.

## Introduction

The importance of cross-disease communication wherein diagnosis of a chronic condition accelerates the risk of a subsequent distinct condition is gaining increased attention.^1,2^ In cancer, preclinical studies demonstrate that induction or presence of specific cardiac events (ie, heart failure,^3^ myocardial infarction^4^) accelerates tumor growth and metastasis in mouse models of intestinal cancer and breast cancer. Related clinical epidemiological data show individuals diagnosed with heart failure or myocardial infarction have increased risk of any incident cancer compared with individuals without these diseases.^5,6,7^ While intriguing, the association between comorbid conditions and cancer outcomes is inconclusive due to a small number of studies that included a small number of incident cancer events and relatively short follow-up periods. Additionally, an unaddressed limitation of prior epidemiological studies is that patients diagnosed with heart failure and myocardial infarction may be more likely to undergo regular screening. Hence, the apparent higher risk of cancer may simply reflect earlier diagnosis.^8^ Finally, to our knowledge, all studies to date have only evaluated cardiac-specific conditions in isolation, the association with a broader range of comorbid conditions categorized into specific classifications on cancer outcomes has not been investigated.

Accordingly, we leveraged data from the Prostate, Lung, Colorectal, and Ovarian (PLCO) screening trial^9,10,11^ to examine the association between comorbid conditions in midlife with cancer incidence and mortality in 128 999 adults without a history of cancer. The goals of investigating this question in PLCO are to address several current knowledge gaps due to the large, nationwide cohort with uniform screening assessments (across participants allocated to the intervention group), long-term follow-up, and rigorous ascertainment and adjudication of all incident cancers and cancer mortality.

## Methods

### PLCO Cohort, Patients, and Setting

Details of the PLCO study design, methods, and cohort characteristics have been reported previously.^9,10,11^ In brief, the PLCO cancer screening trial was designed to evaluate the effects of annual screening (intervention) on cancer mortality, conducted between November 1993 and July 2001. The PLCO protocol was approved by the institutional review board at each participating center and all participants provided written informed consent. Race was classified by the participants using options provided by the investigative team of the PLCO screening trial (American Indian, Asian or Pacific Islander, Black [non-Hispanic], Hispanic, White [non-Hispanic], and unknown). This study followed the Strengthening the Reporting of Observational Studies in Epidemiology (STROBE) reporting guideline. It was not appropriate or possible to involve participants or the public in the design, or conduct, or reporting, or dissemination plans of our research.

At PLCO enrollment, comorbidity history was collected via self-report with participants being asked whether they had a history of any of the following distinct conditions: arthritis, chronic bronchitis, colon-related comorbidities (ulcerative colitis, Crohn disease, Gardner syndrome, familial polyposis), diabetes, diverticulitis or diverticulosis, emphysema, gallbladder stones or inflammation, cardiovascular disease (coronary heart disease or heart attack), hypertension, liver-related comorbidities (hepatitis or cirrhosis), osteoporosis, colorectal polyps or stroke; body mass index (BMI [calculated as weight in kilograms divided by height in meters squared]) at study entry was also assessed. For this analysis, conditions were classified into 1 of 5 specific combinations guided by the World Health Organization’s standard taxonomy system: (1) cardiovascular conditions: coronary heart disease or heart attack, stroke, and hypertension; (2) gastrointestinal conditions: ulcerative colitis, Crohn disease, Gardner syndrome, familial adenomatous polyposis, diverticulitis or diverticulosis, and gallbladder stones or inflammation; (3) respiratory conditions: chronic bronchitis or emphysema; (4) liver conditions: hepatitis or cirrhosis; and (5) metabolic conditions: obesity (BMI ≥30) or diabetes. Arthritis, osteoporosis, and polyps were not evaluated. Of the 154 887 participants enrolled into PLCO, 22 446 participants were excluded from this analysis due to age outside the PLCO entry criteria (n = 36), a history of cancer (n = 6863), history of colorectal polyps (n = 9597), or unknown history of cancer (n = 4930) or polyps (n = 1020). Furthermore, 3442 were excluded due to missing comorbidity data, resulting in a final analytic cohort of 128 999 (eFigure in Supplement 1). Compared with excluded participants (n = 25 888), those included in this analysis were more likely to have been randomized to the intervention group (51% [65 335 of 128 999] vs 47% [12 108 of 25 888]), be younger (median [IQR] age at enrollment: 62 [58-66] vs 63 [59-68] years), male (50% [64 171 of 128 999] vs 48% [12 507 of 25 888]), have fewer smoking pack-years (median [IQR]: 3 [0-31] vs 7 [0-36] years), and less likely to have a history of comorbid conditions at PLCO enrollment.

### Follow-Up, Ascertainment of Incident Cancers, and Cancer Death

PLCO trial participants were contacted annually to ascertain and confirm cancer diagnoses and deaths. This was supplemented by periodic linkage to the National Death Index to enhance completeness of end point ascertainment. Death certificates were obtained to confirm the death. Cause of death was defined based on the National Center for Health Statistics guidance. The trial also used an end-point adjudication process to assign the cause of death in a uniform and unbiased manner.^12,13^ All deaths from causes that were potentially related to one of the PLCO cancers (ie, prostate, lung, colorectal, or ovarian) were reviewed in a blinded manner. The last follow-up of cancer ascertainment in the PLCO was conducted in 2017; the last follow-up for mortality was conducted in 2018. The primary end point was incidence of all cancers combined, defined as a confirmed diagnosis of any invasive cancer during study follow-up.^10^ Secondary end points were incidence of individual cancer types and cancer mortality.

### Statistical Analysis

For analyses of cancer incidence, age was used as the time scale and participants entered the risk set at the time of PLCO study enrollment to account for delayed entry criteria. The cumulative incidence of cancer was estimated using the Aalen-Johanssen method.^14^ For analysis of incidence of all cancers combined, death was considered a competing event; for individual cancer types, other cancers and death were considered competing events. Cox proportional hazards models were used to estimate the cause-specific hazard of cancer. Participants dying without cancer or alive at the end of follow-up were censored. This approach was repeated for the 18 individual cancer types with at least 100 events (male breast cancer not evaluated separately); in these analyses, participants without the specific cancer type being evaluated were also censored. Analysis of cancer mortality was based on the time from cancer diagnosis to death or last known alive date. In the cause-specific Cox models, participants dying of any cancer (not necessarily the same cancer type as their incident diagnosis) were considered to have an event, individuals not dying from cancer or who were alive at the end of follow-up were censored. Proportional hazards were assessed using weighted score tests and visual inspection of survival curves by comorbidity classifications.^15^ Cumulative incidence estimates alongside multivariable cause-specific hazard ratios (HR) and 95% CI are presented.

Median (IQR) follow-up is reported among individuals alive at the end of PLCO follow-up period. Univariable analyses were performed to examine potential associations between relevant clinical covariates and time-to-event end points. Covariates included age at study entry, sex, race and ethnicity, pack-years of cigarette smoking, and PLCO randomization year. Covariates significant in univariable analyses at a threshold of *P* ≤ .20 were included in multivariable models. Age at completion study entry and PLCO randomization group were included in all models. Lifestyle factors such as exercise and alcohol intake at PLCO enrollment were only available for a subset of participants randomized to the intervention group (n = 54 548). Two-sided *P* < .05 was considered statistically significant. Analyses were performed from June 2023 to December 2024 in R version 4.1.2 (R Project for Statistical Computing).^16^

## Results

The characteristics of the 128 999 participants are presented in the Table; 330 (0.3%) were American Indian, 5414 (4.2%) were Asian or Pacific Islander, 6704 (5.2%) were non-Hispanic Black, and 114 073 (88.4%) were non-Hispanic White; 64 171 (49.7%) were male; and the median (IQR) age was 62 (58-66) years. Of these, 49 552 (38.4%) had a history of cardiovascular conditions, 35 652 (27.6%) metabolic conditions, 21 163 (16.4%) gastrointestinal conditions, 8008 (6.2%) respiratory conditions, and 4576 (3.5%) liver conditions. A total of 19 516 participants (15.1%) had a history of co-occurring cardiovascular and metabolic conditions; concurrence of other conditions was infrequent (data not presented). During a median (IQR) follow-up of 20 (19-22) years, 32 857 incident invasive cancers were documented spanning more than 20 cancer types (eTables 1 and 2 in Supplement 1).

**Table.** Characteristics of Participants

Characteristic	Participants, No. (%) (N = 128 999)
Randomization year	
1993	445 (0.3)
1994	12 705 (9.8)
1995	19 559 (15.2)
1996	24 021 (18.6)
1997	21 677 (16.8)
1998	18 335 (14.2)
1999	19 177 (14.9)
2000	10 662 (8.3)
2001	2418 (1.9)
Randomization	
Control	63 664 (49)
Intervention	65 335 (51)
Age at PLCO enrollment, median (IQR), y	62 (58-66)
Sex	
Female	64 828 (50.3)
Male	64 171 (49.7)
Race and ethnicity	
American Indian	330 (0.3)
Asian or Pacific Islander	5414 (4.2)
Black, non-Hispanic	6704 (5.2)
Hispanic	2421 (1.9)
White, non-Hispanic	114 073 (88.4)
Unknown	57 (<0.1)
Pack-years	3 (0-31)
Unknown, No.	1496
History of comorbidities	
Cardiovascular	49 552 (38)
Metabolic	35 652 (28)
Gastrointestinal	21 163 (16.4)
Respiratory	8008 (6.2)
Liver	4576 (3.5)

Abbreviation: PLCO, Prostate, Lung, Colorectal, and Ovarian screening trial.

### Cancer Incidence

**Figure 1.**
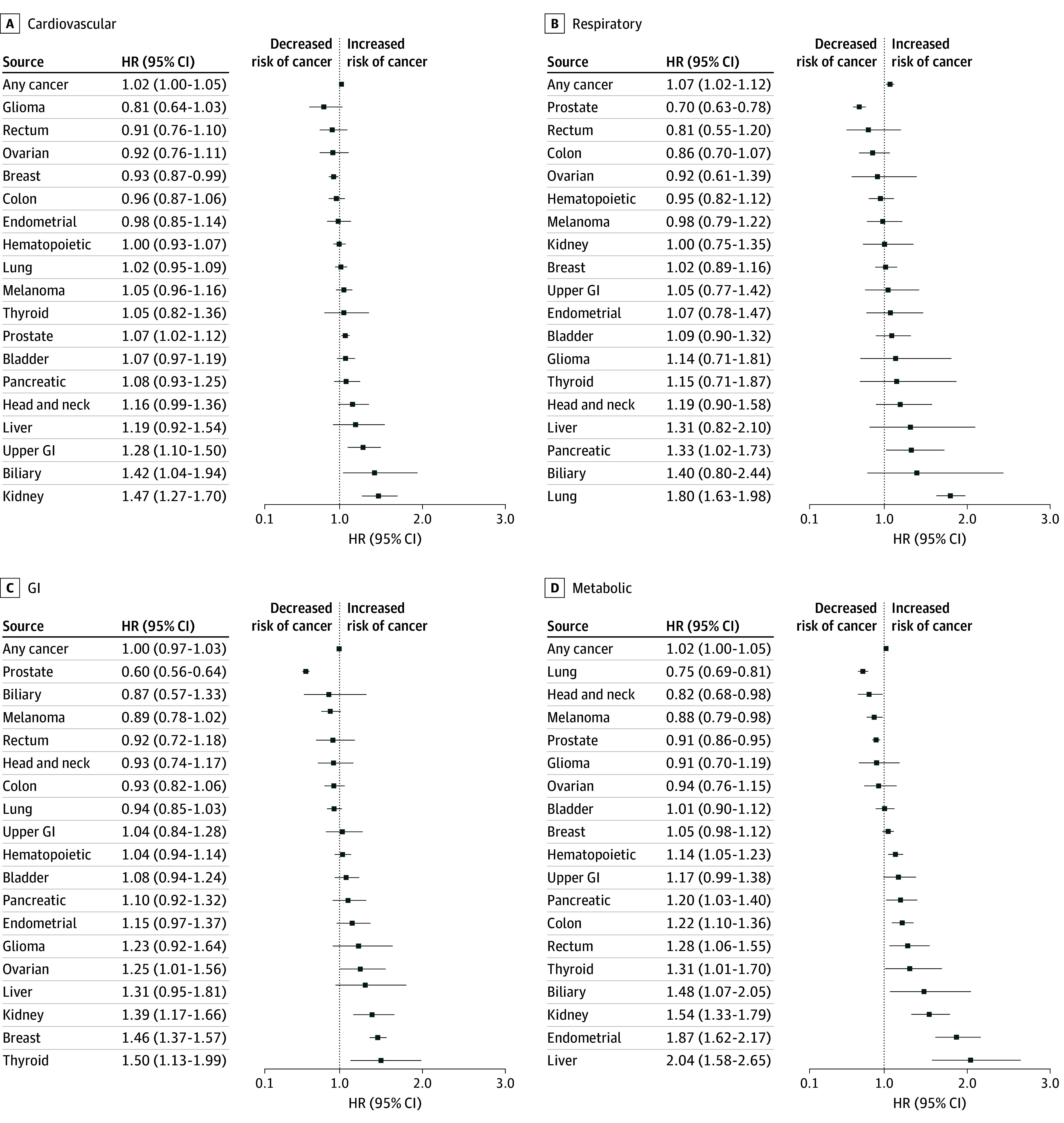
Association of Comorbidity Classifications and Cancer Incidence Cause-specific hazard ratios (HRs) and 95% CIs for risk of any cancer and individual cancer types for: A, history of cardiovascular conditions; B, history of respiratory conditions; C, history of gastrointestinal (GI) conditions; and D, history of metabolic conditions.

In pan-cancer analysis, the risk of any incident cancer was significantly higher for individuals with a history of respiratory conditions (HR, 1.07 [95% CI, 1.02-1.12]) (Figure 1B) and cardiovascular conditions (HR, 1.02 [95% CI, 1.00-1.05]) (Figure 1A). No other individual comorbidity classifications had significant associations. The interaction term for participants with co-occurring cardiovascular and metabolic comorbidities was also not significant (HR, 0.99 [95% CI, 0.94-1.04]).

**Figure 2.**
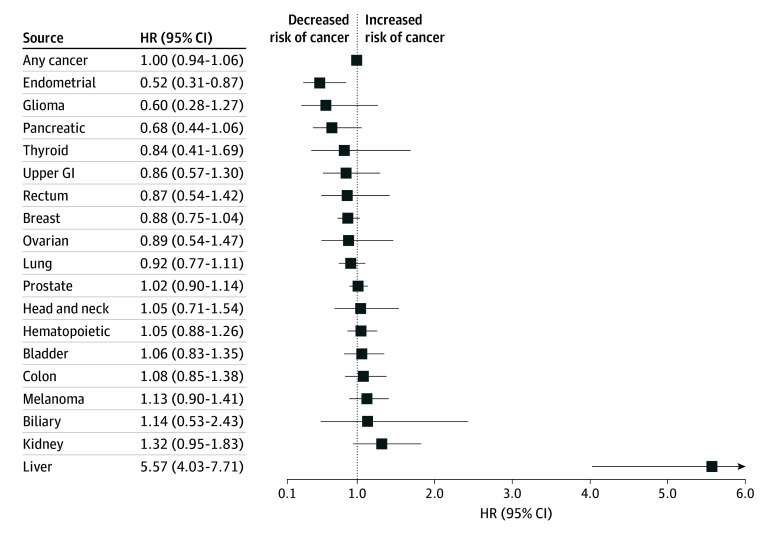
Association of History of Liver Conditions and Cancer Incidence Cause-specific hazard ratios (HRs) and 95% CIs for risk of any cancer and individual cancer types for history of liver conditions. GI indicates gastrointestinal.

For individual cancer types, several significant associations were observed (Figure 1 and Figure 2). Cardiovascular conditions were associated with increased risk of kidney (HR, 1.47 [95% CI, 1.27-1.70]), biliary (HR, 1.42 [95% CI, 1.04-1.94]), upper gastrointestinal (HR, 1.28 [95% CI, 1.10-1.50]), and prostate cancer (HR, 1.07 [95% CI, 1.02-1.12]), and decreased risk of breast cancer (HR, 0.93 [95% CI, 0.87-0.99]) (Figure 1A). Metabolic conditions were associated with increased risk of liver (HR, 2.04 [95% CI, 1.58-2.65]), endometrial (HR, 1.87 [95% CI, 1.62-2.17]), kidney (HR, 1.54 [95% CI, 1.33-1.79]), biliary (HR, 1.48 [95% CI, 1.07-2.05]), thyroid (HR, 1.31 [95% CI, 1.01-1.70]), rectal (HR, 1.28 [95% CI, 1.06-1.55]), colon (HR, 1.22 [95% CI, 1.10-1.36]), pancreas (HR, 1.20 [95% CI, 1.03-1.40]), and hematopoietic cancer (HR, 1.14 [95% CI, 1.05-1.23]), and decreased risk of lung cancer (HR, 0.75 [95% CI, 0.69-0.81]), head and neck cancer (HR, 0.82 [95% CI, 0.68-0.98]), melanoma (HR, 0.88 [95% CI, 0.79-0.98]), and prostate cancer (HR, 0.91 [95% CI, 0.86-0.95]) (Figure 1D).

Gastrointestinal conditions were associated with increased risk of thyroid cancer (HR 1.50 [95% CI, 1.13-1.99]), breast cancer (HR, 1.46 [95% CI, 1.37-1.57]), kidney cancer (HR, 1.39 [95% CI, 1.17-1.66]), and ovarian cancer (HR, 1.25 [95% CI, 1.01-1.56]), and decreased risk of prostate cancer (HR, 0.60 [95% CI, 0.56-0.64]) (Figure 1C). Respiratory conditions were associated with increased risk of lung cancer (HR, 1.80 [95% CI, 1.63-1.98]) and pancreatic cancer (HR, 1.33 [95% CI, 1.02-1.73]), and decreased risk of prostate cancer (HR, 0.70 [95% CI, 0.63-0.78]) (Figure 1B). Finally, liver conditions were associated with increased risk of liver cancer (HR, 5.57 [95% CI, 4.03-7.71]), and decreased risk of endometrial cancer (HR, 0.52 [95% CI, 0.31-0.87]) (Figure 2).

### Cancer Mortality

**Figure 3.**
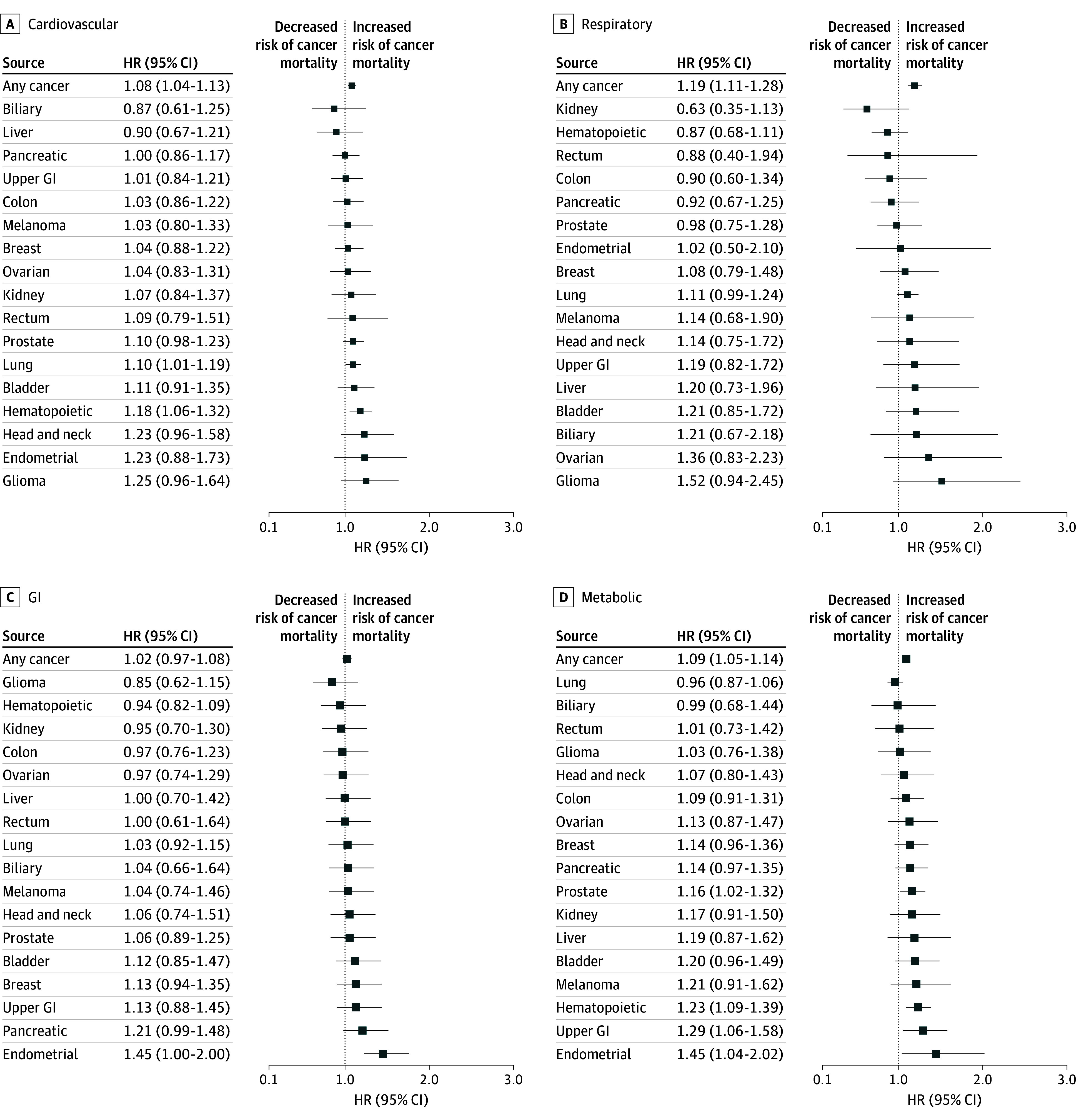
Association of Comorbidity Classifications and Cancer Mortality Cause-specific hazard ratios (HRs) and 95% CIs for risk of any cancer and individual cancer types for: A, history of cardiovascular conditions; B, history of respiratory conditions; C, history of gastrointestinal (GI) conditions; and D, history of metabolic conditions.

The median (IQR) time between cancer diagnosis and last follow-up was 11.1 (6.8-15.6) years. During this period, a total of 17 698 deaths (53.9%) from any cause were documented among the 32 857 participants diagnosed with cancer. Of these, 11 207 (63.3%) were attributed to cancer (eTable 3 in Supplement 1). For analysis of death from any cancer, history of respiratory (HR, 1.19 [95% CI, 1.11-1.28]), cardiovascular (HR, 1.08 [95% CI, 1.04-1.13), and metabolic (HR, 1.09 [95% CI, 1.05-1.14]) conditions were associated with a higher hazard of cancer death compared with individuals diagnosed with cancer without a history of these comorbidities (Figure 3). The interaction term for participants with co-occurring cardiovascular and metabolic comorbidities was not significant (HR, 1.01 [95% CI, 0.93-1.10]).

**Figure 4.**
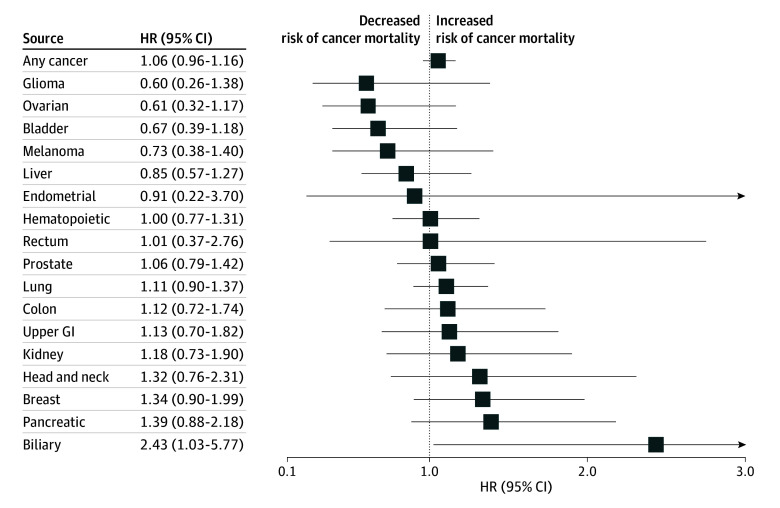
Association of History of Liver Conditions and Cancer Mortality Cause-specific hazard ratios (HRs) and 95% CIs for risk of any cancer and individual cancer types for history of liver conditions. GI indicates gastrointestinal.

For individual cancer types, metabolic conditions were associated with a higher hazard of cancer-specific mortality following a diagnosis of endometrial cancer (HR, 1.45 [95% CI, 1.04-2.02]), upper gastrointestinal cancer (HR, 1.29 [95% CI, 1.06-1.58]), hematopoietic cancer (HR, 1.23 [95% CI, 1.09-1.39]), and prostate cancer (HR, 1.16 [95% CI, 1.02-1.32]) (Figure 3D), whereas cardiovascular conditions were associated with a higher hazard of death following a diagnosis of hematopoietic cancer (HR, 1.18 [95% CI, 1.06-1.32]) and lung cancer (HR, 1.10 [95% CI, 1.01-1.19]) (Figure 3A). No other comorbidity history was associated with cancer death (Figure 3 and Figure 4). Sensitivity analyses adjusting for exercise and alcohol (and other covariates included in the primary analyses) were generally similar to the full analysis (eTables 4 and 5 in Supplement 1).

## Discussion

Cancer pathogenesis is determined by the dynamic interaction between host genetics, somatic genotypes and phenotypes, and the local microenvironment (TME). The TME is exquisitely responsive to alterations in the systemic (host) milieu, leading to complex and dynamic cancer-TME interactions.^17,18^ In this context, findings of the present study suggest the underlying mechanisms of how certain comorbidities alter the systemic milieu and interorgan communication to drive tumorigenesis in different organs is distinct and complex. This notion is highlighted by our pan-cancer analysis showing that only 2 comorbid classifications (ie, respiratory and cardiovascular conditions), while significant, had a relatively small positive association with total cancer risk whereas each comorbid classification evaluated was associated with risk of at least 1 cancer type, with the direction of the association differing as a function of cancer type.

The complexity of the comorbidity-cancer link is illustrated by the association of metabolic conditions (ie, obesity or type 2 diabetes). Metabolic conditions were associated with higher risk of 9 different cancer types, with corresponding HRs ranging from 2.04 (liver) to 1.14 (hematologic). These findings are not unexpected given the well-established positive association of obesity and type 2 diabetes with multiple cancer types^19^ and mechanistic studies demonstrating how dysregulated systemic metabolic control promotes malignant transformation and progression at the TME and cellular levels.^20,21^ The strong positive associations together with the high global prevalence of obesity and type 2 diabetes further underscores the public health importance of efforts to curtail these conditions.^22^ By contrast, metabolic conditions also were associated with lower risk of 4 different cancer types: lung, head and neck, melanoma, and prostate cancer. The reason for these counterintuitive findings is not immediately apparent but may be explained by detection bias (eg, certain cancers are harder to detect in obese individuals) and/or anticancer effects of agents used to manage these conditions (eg, metformin) or other associated conditions (eg, hypertension or hyperlipidemia). The lower risk of melanoma and prostate cancer could be further explained by the confounding influence of certain lifestyle behaviors: exercise is positively associated with risk of melanoma and prostate cancer^23^ and individuals with comorbid conditions likely exercise less compared with those without comorbidities.

Several additional findings merit discussion. First, it is noteworthy that having a history of 3 comorbid classifications evaluated was associated with lower risk of prostate cancer. The potential explanation of healthy screening bias—that men with comorbidities are less likely to undergo routine screening and hence, less likely to be diagnosed—seems unlikely since a high proportion of men were undergoing uniform prostate cancer screening due to randomization to the intervention group in the PLCO trial. Second, our findings showing cardiovascular conditions, including myocardial infarction, were associated with higher risk of 4 different cancer types and corroborate findings of our prior cohort studies showing a diagnosis of either heart failure or myocardial infarction was strongly associated with higher cancer incidence among adults without a history of cancer.^5,6,7^ Of note, cardiovascular conditions also were associated with lower risk of breast cancer, which is contrary to recent work.^4^ Third, perhaps unexpectedly, the co-occurrence of 2 comorbid conditions (ie, cardiovascular and metabolic) was not associated with poorer cancer outcomes vs either one alone, although we did not assess specific combinations of conditions. Fourth, although our data showed the complexity of the comorbidity–cancer incidence relationship in certain scenarios, some were more intuitive: the strongest association observed in the present analysis was association of liver conditions (ie, hepatitis or cirrhosis) and elevated risk of liver cancer, whereas respiratory conditions (ie, chronic bronchitis or emphysema) were strongly associated with elevated risk of lung cancer. Overall, our findings are hypothesis-generating and inform basic and translational studies aiming to elucidate the molecular mechanisms by which select comorbidities promote or suppress the pathogenesis of certain cancer types. Finally, several comorbidity classifications were associated, albeit relatively weakly, with elevated risk of cancer-specific death following a diagnosis of any cancer; the higher hazard of cancer death among individuals with a history of comorbidity may be explained either by selection of suboptimal planned cancer therapy due to concerns regarding tolerability and/or receipt of suboptimal dosing of (optimal) planned therapy due to poor tolerability, however we were unable to adjust for treatment for most cancer types as this information was not collected as part of PLCO, which is an important limitation.

From a clinical perspective, our findings support the incorporation of formal comorbidity screening and/or risk assessment as a routine aspect of cancer screening visits and could be more systematically incorporated into cancer risk calculators. All individuals with a history of relevant comorbid conditions might then receive a program of individualized primary prevention concordant with American Heart Association guidelines.^24^ For instance, exercise is associated with significant reductions in the risk of multiple cancer types in adults without as well as those with a history of comorbid conditions.^23,25^ More aggressive screening of adults with a history of multimorbidity could also be considered, although further research would be required. The potential importance of comorbidities on cancer mortality also supports integration of formal comorbidity screening and/or risk assessment and individualized primary prevention among individuals in which an early lesion is detected (positive test result).^26,27^

### Limitations

This study has limitations that require consideration when interpreting the present findings. First, self-report of comorbidities without medical record verification could be unreliable, and assessment was limited to select conditions likely resulting in relevant conditions not being captured. Exclusion criteria could have introduced selection bias. Timing of diagnosis of each condition relative to PLCO enrollment and important prognostic variables such as cancer stage, grade, and treatment details were not available for all cancers. Second, our analysis was performed among adults voluntarily participating in a cancer screening trial, which may limit generalizability. Indeed, only approximately 11% of participants were from underrepresented racial groups, which does not reflect the proportion across the US. As such, our results are not generalizable to underrepresented racial groups. Additionally, observational studies are susceptible to residual confounding. We adjusted all analyses for available important clinical covariates that may alter the comorbidity–cancer outcomes association as well as performed sensitivity analyses that further adjusted for multiple lifestyle factors (eg, alcohol, exercise) in a subset of the cohort; nevertheless, we were unable to adjust for cancer treatment (outside of the PLCO cancers).

## Conclusion

In this cohort study of 128 999 adults without a history of cancer, comorbid conditions at midlife were associated with risk of total cancer and strongly associated with risk of multiple individual cancer types, with the direction of association differing across cancer types. Our findings may inform clinical management of patients at risk for cancer as well as generate hypotheses that may help guide basic and translational studies elucidating the effects and mechanisms of disease crosstalk in tumorigenesis.

## Notes

Supplement 1. **eFigure.** Flow of Participants **eTable 1.** N Cancer Diagnoses by Comorbidity History **eTable 2.** Cumulative Incidence of Cancer Mortality by History of Multimorbidity Status **eTable 3.** N Cancer Deaths by Cancer Type and Comorbidity History **eTable 4.** Sensitivity Analysis of Cancer Incidence **eTable 5.** Sensitivity Analysis of Cancer-Specific Survival

Supplement 2.
Data Sharing Statement
