# Supplementary material for: Comorbidity in Midlife and Cancer Outcomes
Source: JAMA Netw Open. 2025 Apr 7;8(4):e253469. doi: 10.1001/jamanetworkopen.2025.3469 (PMC11976491; doi:10.1001/jamanetworkopen.2025.3469)
Supplement: Supplement 1. — eFigure. Flow of Participants eTable 1. N Cancer Diagnoses by Comorbidity History eTable 2. Cumulative Incidence of Cancer Mortality by History of Multimorbidity Status eTable 3. N Cancer Deaths by Cancer Type and Comorbidity History eTable 4. Sensitivity Analysis of Cancer Incidence eTable 5. Sensitivity Analysis of Cancer-Specific Survival [file jamanetwopen-e253469-s001.pdf]

Supplemental Online Content

Lavery JA, Boutros PC, Moskowitz CS, Jones LW. Comorbidity in midlife and cancer outcomes. *JAMA Netw Open*. 2025;8(4):e253469. doi:10.1001/jamanetworkopen.2025.3469

eFigure. Flow of Participants

eTable 1. N Cancer Diagnoses by Comorbidity History

eTable 2. Cumulative Incidence of Cancer Mortality by History of Multimorbidity Status

eTable 3. N Cancer Deaths by Cancer Type and Comorbidity History

eTable 4. Sensitivity Analysis of Cancer Incidence

eTable 5. Sensitivity Analysis of Cancer-Specific Survival

This supplemental material has been provided by the authors to give readers additional information about their work.

© 2025 Lavery JA et al. *JAMA Network Open*.

eTable 1. N Cancer Diagnoses by Comorbidity History

Cancer Type	History of Cardiovascular Comorbidities		History of Gastrointestinal Comorbidities		History of Liver Comorbidities		History of Metabolic Comorbidities		History of Respiratory Comorbidities	
	Absent	Present	Absent	Present	Absent	Present	Absent	Present	Absent	Present
Any Cancer*	20125	12732	27718	5139	31665	1192	23983	8874	30755	2102
Biliary	88	85	146	27	166	7	113	60	159	14
Bladder	974	688	1409	253	1594	68	1209	453	1543	119
Breast	3183	1710	3807	1086	4743	150	3552	1341	4640	253
Colon	1086	687	1488	285	1705	68	1243	530	1681	92
Endometrial	506	316	660	162	806	16	501	321	780	42
Glioma	219	102	262	59	314	7	246	75	302	19
Head & Neck	396	281	589	88	650	27	511	166	620	57
Hematopoietic	2028	1248	2735	541	3152	124	2360	916	3104	172
Liver	131	123	205	49	209	45	141	113	234	20
Lung	2043	1387	2901	529	3306	124	2612	818	2891	539
Melanoma	1297	718	1745	270	1934	81	1557	458	1925	90
Ovarian	338	181	416	103	502	17	393	126	494	25
Pancreatic	477	340	669	148	796	21	569	248	753	64
Prostate	5243	3271	7623	891	8214	300	6461	2053	8152	362
Rectum	337	202	463	76	522	17	367	172	512	27
Renal	397	389	620	166	748	38	488	298	737	49
Thyroid	176	105	212	69	273	8	186	95	263	18
Upper GI	368	333	596	105	678	23	483	218	655	46

* Any cancer includes the cancer types shown on the table in addition to male breast and unspecified sites.

© 2025 Lavery JA et al. *JAMA Network Open*.

eTable 2. Cumulative Incidence of Cancer Mortality by History of Multimorbidity Status

	Years post-diagnosis			
	**5**	**10**	**15**	**20**
**Cardiovascular**				
Absent	25% (25%-26%)	31% (30%-32%)	35% (35%-36%)	39% (39%-40%)
Present	28% (28%-29%)	34% (33%-35%)	38% (37%-39%)	41% (40%-42%)
**Gastrointestinal**				
Absent	26% (26%-27%)	32% (31%-33%)	36% (36%-37%)	40% (39%-41%)
Present	27% (26%-29%)	33% (32%-34%)	38% (36%-39%)	41% (39%-42%)
**Respiratory**				
Absent	26% (25%-26%)	32% (31%-32%)	36% (35%-36%)	39% (39%-40%)
Present	36% (34%-38%)	42% (40%-44%)	46% (44%-48%)	48% (46%-51%)
**Liver**				
Absent	26% (26%-27%)	32% (32%-33%)	36% (36%-37%)	40% (39%-41%)
Present	29% (27%-32%)	35% (33%-38%)	39% (36%-42%)	41% (38%-45%)
**Metabolic**				
Absent	26% (25%-26%)	31% (31%-32%)	36% (35%-36%)	39% (39%-40%)
Present	29% (28%-30%)	34% (33%-35%)	38% (37%-39%)	41% (40%-42%)

© 2025 Lavery JA et al. *JAMA Network Open*.

eTable 3. N Cancer Deaths by Cancer Type and Comorbidity History

6

Cancer Type	History of Cardiovascular Comorbidities		History of Gastrointestinal Comorbidities		History of Liver Comorbidities		History of Metabolic Comorbidities		History of Respiratory Comorbidities	
	Absent	Present	Absent	Present	Absent	Present	Absent	Present	Absent	Present
Any Cancer*	6622	4585	9391	1816	10767	440	8023	3184	10283	924
Biliary	74	65	114	25	133	6	90	49	126	13
Bladder	234	194	362	66	415	13	303	125	391	37
Breast	415	246	502	159	635	26	470	191	616	45
Colon	379	236	531	84	592	23	422	193	588	27
Endometrial	95	68	124	39	161	2	90	73	155	8
Glioma	198	93	240	51	285	6	228	63	272	19
Head & Neck	145	117	226	36	248	14	197	65	236	26
Hematopoietic	827	570	1165	232	1339	58	975	422	1327	70
Liver	106	101	163	44	175	32	115	92	188	19
Lung	1556	1074	2238	392	2533	97	2001	629	2229	401
Melanoma	167	108	234	41	265	10	209	66	258	17
Ovarian	233	120	279	74	343	10	269	84	334	19
Pancreatic	429	299	598	130	707	21	508	220	678	50
Prostate	784	526	1152	158	1260	50	981	329	1251	59
Rectum	115	72	164	23	182	5	124	63	180	7
Renal	148	135	227	56	264	19	168	115	270	13
Thyroid	21	12	29	4	33	0	23	10	32	1
Upper GI	253	233	411	75	468	18	328	158	454	32

* Any cancer includes the cancer types shown on the table in addition to male breast and unspecified sites.

© 2025 Lavery JA et al. *JAMA Network Open*.

eTable 4. Sensitivity Analysis of Cancer Incidence

Cancer Type	Cardiovascular Disease Related		Respiratory Related		Gastrointestinal Related		Liver Related		Metabolic Related	
	Primary	Sensitivity	Primary	Sensitivity	Primary	Sensitivity	Primary	Sensitivity	Primary	Sensitivity
All Cancers	1.02 (1.00, 1.05)	1.06 (1.02, 1.10)	1.07 (1.02, 1.12)	1.05 (0.98, 1.13)	1.00 (0.97, 1.03)	1.02 (0.97, 1.07)	1.00 (0.94, 1.06)	0.96 (0.88, 1.05)	1.02 (1.00, 1.05)	1.01 (0.98, 1.06)
Biliary	1.42 (1.04, 1.94)	1.18 (0.73, 1.91)	1.40 (0.80, 2.44)	1.45 (0.62, 3.41)	0.87 (0.57, 1.33)	1.09 (0.59, 2.02)	1.14 (0.53, 2.43)	1.96 (0.79, 4.88)	1.48 (1.07, 2.05)	1.15 (0.68, 1.93)
Bladder	1.07 (0.97, 1.19)	1.04 (0.89, 1.22)	1.09 (0.90, 1.32)	1.01 (0.74, 1.37)	1.08 (0.94, 1.24)	1.11 (0.90, 1.36)	1.06 (0.83, 1.35)	0.87 (0.58, 1.32)	1.01 (0.90, 1.12)	1.06 (0.90, 1.26)
Breast	0.93 (0.87, 0.99)	0.92 (0.84, 1.01)	1.02 (0.89, 1.16)	0.92 (0.75, 1.13)	1.46 (1.37, 1.57)	1.53 (1.38, 1.70)	0.88 (0.75, 1.04)	0.87 (0.67, 1.11)	1.05 (0.98, 1.12)	0.96 (0.87, 1.06)
Colon	0.96 (0.87, 1.06)	1.04 (0.89, 1.22)	0.86 (0.70, 1.07)	0.66 (0.44, 0.98)	0.93 (0.82, 1.06)	1.00 (0.82, 1.23)	1.08 (0.85, 1.38)	1.19 (0.82, 1.74)	1.22 (1.10, 1.36)	1.37 (1.16, 1.63)
Endometrial	0.98 (0.85, 1.14)	1.08 (0.86, 1.35)	1.07 (0.78, 1.47)	1.14 (0.70, 1.83)	1.15 (0.97, 1.37)	1.11 (0.85, 1.45)	0.52 (0.31, 0.87)	0.41 (0.17, 0.99)	1.87 (1.62, 2.17)	1.82 (1.44, 2.29)
Glioma	0.81 (0.64, 1.03)	1.00 (0.70, 1.42)	1.14 (0.71, 1.81)	1.17 (0.57, 2.39)	1.23 (0.92, 1.64)	1.37 (0.90, 2.10)	0.60 (0.28, 1.27)	0.39 (0.10, 1.58)	0.91 (0.70, 1.19)	0.86 (0.57, 1.30)
Head & Neck	1.16 (0.99, 1.36)	0.99 (0.78, 1.27)	1.19 (0.90, 1.58)	1.56 (1.04, 2.33)	0.93 (0.74, 1.17)	0.82 (0.57, 1.18)	1.05 (0.71, 1.54)	1.06 (0.58, 1.93)	0.82 (0.68, 0.98)	0.93 (0.71, 1.22)
Hematopoietic	1.00 (0.93, 1.07)	1.05 (0.94, 1.17)	0.95 (0.82, 1.12)	0.88 (0.68, 1.13)	1.04 (0.94, 1.14)	1.03 (0.89, 1.19)	1.05 (0.88, 1.26)	1.06 (0.81, 1.40)	1.14 (1.05, 1.23)	1.10 (0.98, 1.24)
Liver	1.19 (0.92, 1.54)	0.98 (0.66, 1.45)	1.31 (0.82, 2.10)	1.49 (0.77, 2.91)	1.31 (0.95, 1.81)	1.45 (0.91, 2.31)	5.57 (4.03, 7.71)	5.24 (3.15, 8.71)	2.04 (1.58, 2.65)	2.13 (1.43, 3.17)
Lung	1.02 (0.95, 1.09)	0.94 (0.84, 1.05)	1.80 (1.63, 1.98)	1.83 (1.57, 2.13)	0.94 (0.85, 1.03)	0.86 (0.74, 1.00)	0.92 (0.77, 1.11)	1.06 (0.81, 1.39)	0.75 (0.69, 0.81)	0.71 (0.62, 0.81)

© 2025 Lavery JA et al. *JAMA Network Open*.

Melanoma	1.05 (0.96, 1.16)	1.19 (1.03, 1.37)	0.98 (0.79, 1.22)	1.09 (0.79, 1.49)	0.89 (0.78, 1.02)	0.88 (0.72, 1.07)	1.13 (0.90, 1.41)	0.92 (0.64, 1.34)	0.88 (0.79, 0.98)	0.88 (0.75, 1.04)
Ovarian	0.92 (0.76, 1.11)	1.10 (0.84, 1.44)	0.92 (0.61, 1.39)	0.84 (0.44, 1.59)	1.25 (1.01, 1.56)	1.26 (0.92, 1.73)	0.89 (0.54, 1.47)	0.84 (0.40, 1.78)	0.94 (0.76, 1.15)	1.02 (0.75, 1.37)
Pancreatic	1.08 (0.93, 1.25)	1.09 (0.88, 1.36)	1.33 (1.02, 1.73)	1.43 (0.97, 2.11)	1.10 (0.92, 1.32)	0.93 (0.69, 1.24)	0.68 (0.44, 1.06)	0.97 (0.54, 1.73)	1.20 (1.03, 1.40)	1.19 (0.94, 1.50)
Prostate	1.07 (1.02, 1.12)	1.12 (1.05, 1.20)	0.70 (0.63, 0.78)	0.72 (0.61, 0.85)	0.60 (0.56, 0.64)	0.64 (0.58, 0.71)	1.02 (0.90, 1.14)	0.89 (0.74, 1.07)	0.91 (0.86, 0.95)	0.93 (0.87, 1.01)
Rectum	0.91 (0.76, 1.10)	0.86 (0.63, 1.19)	0.81 (0.55, 1.20)	0.93 (0.49, 1.78)	0.92 (0.72, 1.18)	1.08 (0.72, 1.61)	0.87 (0.54, 1.42)	0.79 (0.32, 1.92)	1.28 (1.06, 1.55)	1.63 (1.18, 2.24)
Renal	1.47 (1.27, 1.70)	1.55 (1.26, 1.91)	1.00 (0.75, 1.35)	1.00 (0.64, 1.57)	1.39 (1.17, 1.66)	1.35 (1.04, 1.74)	1.32 (0.95, 1.83)	1.24 (0.76, 2.02)	1.54 (1.33, 1.79)	1.28 (1.02, 1.60)
Thyroid	1.05 (0.82, 1.36)	1.22 (0.83, 1.81)	1.15 (0.71, 1.87)	1.64 (0.82, 3.29)	1.50 (1.13, 1.99)	1.67 (1.09, 2.56)	0.84 (0.41, 1.69)	1.25 (0.51, 3.08)	1.31 (1.01, 1.70)	1.29 (0.85, 1.94)
Upper GI	1.28 (1.10, 1.50)	1.49 (1.16, 1.90)	1.05 (0.77, 1.42)	0.79 (0.45, 1.40)	1.04 (0.84, 1.28)	1.12 (0.81, 1.57)	0.86 (0.57, 1.30)	0.87 (0.45, 1.69)	1.17 (0.99, 1.38)	1.23 (0.94, 1.60)

© 2025 Lavery JA et al. *JAMA Network Open*.

eTable 5. Sensitivity Analysis of Cancer-Specific Survival

Cancer Type	Cardiovascular Disease Related		Respiratory Related		Gastrointestinal Related		Liver Related		Metabolic Related	
	Primary	Sensitivity	Primary	Sensitivity	Primary	Sensitivity	Primary	Sensitivity	Primary	Sensitivity
All Cancers	1.08 (1.04, 1.13)	1.02 (0.96, 1.08)	1.19 (1.11, 1.28)	1.22 (1.10, 1.36)	1.02 (0.97, 1.08)	1.03 (0.95, 1.12)	1.06 (0.96, 1.16)	1.29 (1.11, 1.48)	1.09 (1.05, 1.14)	1.12 (1.05, 1.20)
Biliary	0.87 (0.61, 1.25)	0.84 (0.47, 1.51)	1.21 (0.67, 2.18)	0.67 (0.24, 1.86)	1.04 (0.66, 1.64)	0.93 (0.45, 1.94)	2.43 (1.03, 5.77)	2.24 (0.75, 6.70)	0.99 (0.68, 1.44)	1.10 (0.58, 2.12)
Bladder	1.11 (0.91, 1.35)	1.13 (0.83, 1.55)	1.21 (0.85, 1.72)	1.23 (0.71, 2.14)	1.12 (0.85, 1.47)	1.23 (0.80, 1.88)	0.67 (0.39, 1.18)	1.07 (0.44, 2.64)	1.20 (0.96, 1.49)	1.22 (0.86, 1.72)
Breast	1.04 (0.88, 1.22)	0.93 (0.71, 1.21)	1.08 (0.79, 1.48)	0.88 (0.51, 1.54)	1.13 (0.94, 1.35)	1.26 (0.96, 1.66)	1.34 (0.90, 1.99)	1.39 (0.77, 2.49)	1.14 (0.96, 1.36)	1.05 (0.78, 1.41)
Colon	1.03 (0.86, 1.22)	0.89 (0.67, 1.18)	0.90 (0.60, 1.34)	0.77 (0.34, 1.74)	0.97 (0.76, 1.23)	0.79 (0.53, 1.18)	1.12 (0.72, 1.74)	1.34 (0.70, 2.55)	1.09 (0.91, 1.31)	1.22 (0.91, 1.64)
Endometrial	1.23 (0.88, 1.73)	1.13 (0.64, 1.99)	1.02 (0.50, 2.10)	0.79 (0.19, 3.35)	1.45 (1.00, 2.10)	0.84 (0.44, 1.62)	0.91 (0.22, 3.70)	0.93 (0.12, 7.21)	1.45 (1.04, 2.02)	1.85 (1.04, 3.27)
Glioma	1.25 (0.96, 1.64)	1.16 (0.77, 1.73)	1.52 (0.94, 2.45)	1.95 (0.91, 4.16)	0.85 (0.62, 1.15)	0.91 (0.57, 1.45)	0.60 (0.26, 1.38)	0.76 (0.17, 3.34)	1.03 (0.76, 1.38)	1.15 (0.72, 1.84)
Head & Neck	1.23 (0.96, 1.58)	1.09 (0.72, 1.64)	1.14 (0.75, 1.72)	1.04 (0.57, 1.92)	1.06 (0.74, 1.51)	1.19 (0.66, 2.15)	1.32 (0.76, 2.31)	1.15 (0.41, 3.23)	1.07 (0.80, 1.43)	1.05 (0.65, 1.68)
Hematopoietic	1.18 (1.06, 1.32)	1.20 (1.02, 1.42)	0.87 (0.68, 1.11)	0.82 (0.55, 1.21)	0.94 (0.82, 1.09)	1.03 (0.83, 1.28)	1.00 (0.77, 1.31)	1.15 (0.77, 1.71)	1.23 (1.09, 1.39)	1.29 (1.08, 1.56)
Liver	0.90 (0.67, 1.21)	0.93 (0.56, 1.52)	1.20 (0.73, 1.96)	1.95 (0.92, 4.12)	1.00 (0.70, 1.42)	0.81 (0.48, 1.38)	0.85 (0.57, 1.27)	0.75 (0.38, 1.51)	1.19 (0.87, 1.62)	0.92 (0.55, 1.55)
Lung	1.10 (1.01, 1.19)	1.03 (0.90, 1.18)	1.11 (0.99, 1.24)	1.10 (0.92, 1.31)	1.03 (0.92, 1.15)	1.03 (0.86, 1.24)	1.11 (0.90, 1.37)	1.28 (0.94, 1.75)	0.96 (0.87, 1.06)	1.07 (0.92, 1.24)

© 2025 Lavery JA et al. *JAMA Network Open*.

Melanoma	1.03 (0.80, 1.33)	0.98 (0.66, 1.45)	1.14 (0.68, 1.90)	1.12 (0.49, 2.60)	1.04 (0.74, 1.46)	1.68 (1.04, 2.70)	0.73 (0.38, 1.40)	1.51 (0.61, 3.77)	1.21 (0.91, 1.62)	1.06 (0.67, 1.68)
Ovarian	1.04 (0.83, 1.31)	0.83 (0.59, 1.15)	1.36 (0.83, 2.23)	1.77 (0.70, 4.44)	0.97 (0.74, 1.29)	1.29 (0.87, 1.92)	0.61 (0.32, 1.17)	0.67 (0.25, 1.80)	1.13 (0.87, 1.47)	1.06 (0.71, 1.59)
Pancreatic	1.00 (0.86, 1.17)	1.05 (0.82, 1.35)	0.92 (0.67, 1.25)	0.84 (0.52, 1.36)	1.21 (0.99, 1.48)	1.48 (1.06, 2.06)	1.39 (0.88, 2.18)	1.45 (0.77, 2.70)	1.14 (0.97, 1.35)	1.21 (0.92, 1.58)
Prostate	1.10 (0.98, 1.23)	1.15 (0.97, 1.37)	0.98 (0.75, 1.28)	1.21 (0.84, 1.75)	1.06 (0.89, 1.25)	0.95 (0.73, 1.23)	1.06 (0.79, 1.42)	1.48 (0.98, 2.24)	1.16 (1.02, 1.32)	1.07 (0.87, 1.30)
Rectum	1.09 (0.79, 1.51)	0.51 (0.24, 1.06)	0.88 (0.40, 1.94)	0.36 (0.04, 3.18)	1.00 (0.61, 1.64)	0.85 (0.30, 2.36)	1.01 (0.37, 2.76)	2.02 (0.26, 15.53)	1.01 (0.73, 1.42)	2.03 (1.01, 4.09)
Renal	1.07 (0.84, 1.37)	1.19 (0.82, 1.72)	0.63 (0.35, 1.13)	0.45 (0.19, 1.07)	0.95 (0.70, 1.30)	0.94 (0.59, 1.51)	1.18 (0.73, 1.90)	1.72 (0.84, 3.52)	1.17 (0.91, 1.50)	1.15 (0.78, 1.71)
Upper GI	1.01 (0.84, 1.21)	0.83 (0.61, 1.12)	1.19 (0.82, 1.72)	1.24 (0.62, 2.47)	1.13 (0.88, 1.45)	1.25 (0.84, 1.86)	1.13 (0.70, 1.82)	1.17 (0.55, 2.50)	1.29 (1.06, 1.58)	1.44 (1.03, 2.01)

16

© 2025 Lavery JA et al. *JAMA Network Open*.

eFigure. Flow of Participants

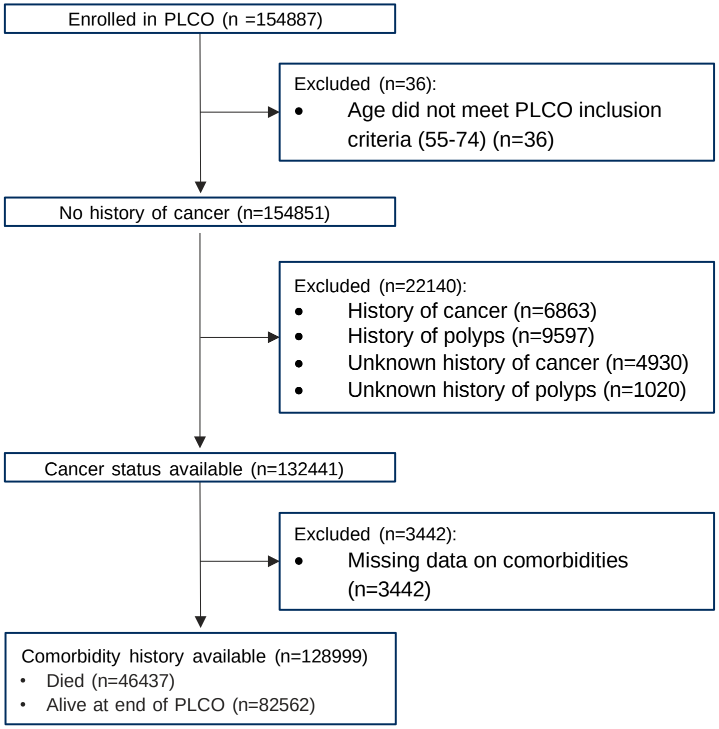

© 2025 Lavery JA et al. *JAMA Network Open*.
