# Supplementary material for: Comorbidity in Midlife and Cancer Outcomes
Source: JAMA Netw Open. 2025 Apr 7;8(4):e253469. doi: 10.1001/jamanetworkopen.2025.3469 (PMC11976491; doi:10.1001/jamanetworkopen.2025.3469)
Supplement: Supplement 2. — Data Sharing Statement [file jamanetwopen-e253469-s002.pdf]

Data Sharing Statement

Lavery. Comorbidity in Midlife and Cancer Outcomes. *JAMA Netw Open*. Published April 07, 2025. doi:10.1001/jamanetworkopen.2025.3469

Data
Data available: Yes
Data types: Deidentified participant data
How to access data: All data is available to the public via the PLCO website
When available: With publication

Supporting Documents
Document types: Statistical/analytic code
How to access documents: will be provided upon reasonable request

Additional Information
Who can access the data: All data is available to the public via the PLCO website
Types of analyses: Upon reasonable request
Mechanisms of data availability: with investigator support
Any additional restrictions: N/A
